# Exploring How Older Adults Experience semAPP, a 360° Media–Based Tool for Memory Assessment: Qualitative Study

**DOI:** 10.2196/56796

**Published:** 2024-12-05

**Authors:** Francesca Bruni, Valentina Mancuso, Jonathan Panigada, Marco Stramba-Badiale, Pietro Cipresso, Elisa Pedroli

**Affiliations:** 1 Department of Theoretical and Applied Sciences eCampus University Novedrate Italy; 2 Department of Geriatrics and Cardiovascular Medicine IRCCS Istituto Auxologico Italiano Milan Italy; 3 Department of Psychology University of Turin Turin Italy

**Keywords:** assessment, virtual reality, 360-degree videos, user experience, memory, aging, psycho gerontology

## Abstract

**Background:**

Technology is already a part of our daily lives, and its influence is growing rapidly. This evolution has not spared the health care field. Nowadays, a crucial challenge is considering aspects such as design, development, and implementation, highlighting their functionality, ease of use, compatibility, performance, and safety when a new technological tool is developed. As noted in many works, the abandonment rate is usually higher when a user has a terrible experience with these instruments. It would be appropriate to incorporate the final users—whether they are patients, health care professionals, or both—in the stages of instrument design to understand their needs and preferences. Since most apps that fail did not include end users and health care professionals in the development phase, their involvement at all stages of app development may increase their commitment and improve integration, self-management, and health outcomes.

**Objective:**

This study aims (1) to develop semAPP (spatial and episodic memory assessment application), a 360° media–based tool, to assess memory in aging by simulating a real-life situation and (2) to test the usability of the app and the connected experience in an end-user population.

**Methods:**

A total of 34 older adults participated in the study: 16 (47%) healthy individuals and 18 (53%) patients with mild cognitive impairment. They used semAPP and completed qualitative and quantitative measures. The app includes 2 parts: object recognition and spatial memory tasks. During the first task, users have to navigate in an apartment freely and visit rooms, and then they must recognize the right map of the house. In the second task, users are immersed in a living room, and they have to encode and then recall some target objects, simulating a relocation. We deployed this app on an 11.2-inch iPad, and we tested its usability and the experience of users interacting with the app. We conducted descriptive analyses for both the entire sample and each subgroup; we also conducted parametric and correlation analyses to compare groups and to examine the relationship between task execution and the virtual experience, as well as the acceptance of technology.

**Results:**

Both groups judged the app as an easy-to-use tool, and they were willing to use it. Moreover, the results match the idea that usability might be influenced by different factors depending on instrument and personal features, such as presentation, functionality, system performance, interactive behavior, attitudes, skills, and personality.

**Conclusions:**

The findings support the possibility of using semAPP in older patients, as well as the importance of designing and evaluating new technological tools, considering not only the general population but also the specific target ones.

## Introduction

Technology integration into our daily lives has become increasingly prevalent and shows no signs of abating. The use of mobile devices and videoconferencing for interpersonal communication among family and friends, as well as the prevalence of programmable household appliances such as the microwave, dishwasher, coffee maker, and oven, serve as illustrative instances of the pervasive presence of technology in our everyday existence. Modern entertainment technology encompasses a variety of sophisticated devices, such as smart televisions equipped with multiple functions, digital video recorders, digital music players, and cameras, among others. These examples illustrate the diverse range of options available in the realm of contemporary entertainment technology. Hence, people are compelled to cultivate additional skills to interact with these technological advancements. Moreover, older people must acclimate themselves to the modifications imposed by the ongoing expansion of technology. One of the key concerns pertains to the provision of training for older individuals to enable them to engage with new technologies securely and proficiently [1]. On one hand, the advancements in technology present significant opportunities for older individuals to derive advantages from these novel innovations. However, many older people hesitate to use technology due to several obstacles, including a lack of assistance, unclear instructions, or a lack of confidence [2]. Generally, age-related problems (eg, the impairment of cognitive abilities), personal perception (eg, computer self-efficacy and anxiety related to the use of technology), and technology-related barriers (eg, interface usability) may influence the experience with technology [3,4]. Even so, the computer tablet is a widely adopted and user-friendly technological solution. The interface exhibits an easy-to-use nature and comparatively lower complexity than alternative interfaces, while also obviating the need for wired infrastructure. Consequently, an increasing number of researchers are using this instrument to provide health care interventions in aging [5]. However, this solution is not exempt from criticism. Vaportzis et al [2] identified several obstacles associated with the use of tablets. The barriers to technology adoption among older individuals include insufficient or overly complex instructions and guidance, limited knowledge or confidence in using devices, concerns about the potential risks associated with technology, health-related obstacles, and the high costs of devices. Additionally, older individuals tend to be slower in adapting to new technologies compared to younger individuals, resulting in lower technology use and less enthusiasm toward its adoption. Notably, the integration of technology into geriatric care is becoming increasingly important, particularly considering the impending shift toward technologically driven cognitive assessment tools.

Technological advancements offer the potential for more precise, efficient, and accessible cognitive assessment tools that can provide real-time data, remote monitoring, and personalized interventions. Traditional assessments are provided through paper-and-pencil tests or computerized tools; however, an open-ended question in the neuropsychological field regards the ecological validity of the employed measures, that is, how to measure cognitive functions reliably and validly [6]. Research suggests that assessment tools do not accurately reflect the demands of the everyday world in predicting cognitive functioning [6,7]. Due to their ability to produce realistic surroundings in a controlled and safe manner, 360° media may be the greatest approach to address this issue and enhance the accuracy of the neuropsychological assessment process [8-11]. In the same way as computer-generated virtual reality games and other interactive experiences are designed to be viewed through headsets, 360° videos and images, sometimes referred to as immersive videos or spherical media, may also be viewed on flat-screen devices like a smartphone or computer by dragging the viewpoint with a mouse or a finger, as well as more immersive devices. Live action in the real world is recorded using special cameras that capture the entire environment. Media are recorded thanks to omnidirectional lenses with a circular fisheye view of the surrounding environment, allowing us to get files of the complete environment. Moreover, they provide the possibility to capture different points of view: by placing the camera on the recorder’s head while a video is being made, the user can obtain a first-person perspective of the action. If not, the user can position the camera anywhere in the scene to view it from the perspective of an outsider (third-person perspective). Because 360° media show the full globe instead of just a small section of it, they are different from 2D videos. Technology and software elements that trick the user into believing they are surrounded by an alternate dimension can also be used to establish a sensation of presence using 360° media [12]. This technology offers several additional advantages including cost-effectiveness, ease of use compared to computer environments, and a user-friendly design [13]. The ability to modify the participant’s position in space and place in a realistic virtual environment also increases the ecological validity of the tests and promotes an embodied experience significantly [14].

Characteristics of 360° media could be attractive for memory testing, boosting the procedure’s precision. Memory plays a central role in various aspects of daily life, such as recalling important information, managing medications, and navigating familiar environments. For older adults, maintaining optimal memory function is essential to ensure their independence, safety, and quality of life. The efficacy or efficiency of processing measures typically show a linear decline with age in the cognitive profile of aging [15], and memory performance, attention, and executive functions are some of the abilities that decline over time [16], risking cognitive impairment when problems are significant. In particular, memory problems may be crucial in predicting the chance of acquiring dementia, such as Alzheimer disease [17,18]. However, the early indications of cognitive impairment are typically ignored because they are confused about the consequences of physiological aging. Thus, a prompt evaluation is the most effective way to ascertain the extent of the problems that distinguish pathological from healthy aging. To stop the progression and avoid disability, it is therefore essential to identify issues swiftly and effectively. Implementing timely neuropsychological evaluations and cutting-edge techniques, such as virtual reality–based approaches that demonstrate enhanced sensitivity for the early detection of cognitive deficits, may represent a promising option [19].

The examination of the challenges that older adults encounter when using technology is crucial to identifying the most efficacious approaches for introducing technology within the clinical domain, given its significant benefits and necessity in this context [20]. The elements that affect older people’s adoption and use of technology must be identified to better understand and anticipate their technology use behavior. One of the most significant indicators of the adoption and use of technology is technology acceptance, which is the attitudinal perception and behavioral desire to use technology [21]. Several models or theories have been proposed to explain technology acceptance behavior; among these, Chen and Chan [21] developed the first theoretical model to predict older people’s acceptance of everyday technology (ie, the senior technology acceptance model), considering individual attributes, gerontechnology self-efficacy, anxiety, health, and ability characteristics. Moreover, a crucial feature to provide an instrument usable and accepted by users is to design the tool based on the target population, to make sure that the scenario will meet the needs, concerns, and expectations listed by individuals. Thus, understanding and addressing the health care needs of older adults have become increasingly crucial. Among these needs, the evaluation of cognitive functions stands out as a fundamental aspect of comprehensive geriatric care. It is well recognized that including clinicians, user experience (UX) experts, and end users allows for the consideration of useful information targeted at creating a suitable interaction between the patient, the technology, and health care organizations [22]. Since most apps that fail did not include end users and health care professionals in the development phase, their involvement could increase their commitment and improve integration, self-management, and health results. However, when a new app is developed, most tests are improperly provided to a general population engaged in evaluating critical aspects, judging their experience, and predicting the adoption of the presented product.

Based on these considerations, we developed a new app to evaluate memory in aging: semAPP (spatial and episodic memory assessment application), testing the connected experience in a population typically characterized by memory problems: mild cognitive impairment (MCI). We compared their experience with that of the healthy population to expand our knowledge of older people’s experience with technology. This study aims to present the UX of a 360° tool designed to assess memory in aging.

## Methods

### Recruitment

A total of 34 older adults (mean age 74.65, SD 7.64 years) were recruited at the Medical Rehabilitation Department of IRCCS (Istituto di Ricovero e Cura a Carattere Scientifico) Istituto Auxologico Italiano in Milan. Patients and outpatients aged 60 years and older from the clinical institution were selected for this study. During their hospitalizations, comprehensive information regarding the research was provided, and participation was entirely voluntary. Similarly, outpatients received the same information during routine clinical visits, allowing them to make an informed decision to participate in the experiment. The entire sample consisted of 15 (44%) male participants and 19 (56%) female participants. All participants were native Italian speakers and took part voluntarily in the study after signing an informed consent form. Based on an initial neuropsychological assessment, participants were divided into 2 groups: healthy participants (n=16, 47%) and patients with MCI (n=18, 53%). The inclusion criteria were (1) aged 60 years and older (without maximum age limitation) and (2) normal or corrected-to-normal vision. Exclusion criteria were (1) invalidating internist, psychiatric, and neurological conditions that could affect the performances and (2) cognitive impairments certifiable by a score lower than 24 points on the Mini-Mental State Examination, Italian version [23,24]. On the other hand, patients with MCI were identified based on self-reported (or reported by a caregiver) cognitive decline, an objective impairment on the neuropsychological testing, preservation in functional abilities, and no evidence of significant impairment in social or occupational functioning (ie, not demented) [17].

### Ethical Considerations

The data collection has been conducted anonymously, according to the Regulation (EU) 2016/679 of the European Parliament and of the Council (General Data Protection Regulation). The study received ethical approval from the Ethical Committee of the IRCCS Istituto Auxologico Italiano (2022_01_25_04) and complies with the ethical principles set out in the Helsinki Declaration. No financial compensation was provided to the participants. All patients provided informed consent for the study and no financial compensation was provided.

### semAPP

#### Overview

semAPP consists of 2 memory tasks created with 360° media aimed to assess memory by simulating a real-life situation. The 2 exercises are focused on episodic and spatial memory, respectively. Both are structured into 2 main phases: learning, in which the user has to memorize specific features, and recalling, in which what was learned in the first phase has to be used to answer new requests.

#### Spatial Memory Task

**Figure 1:**
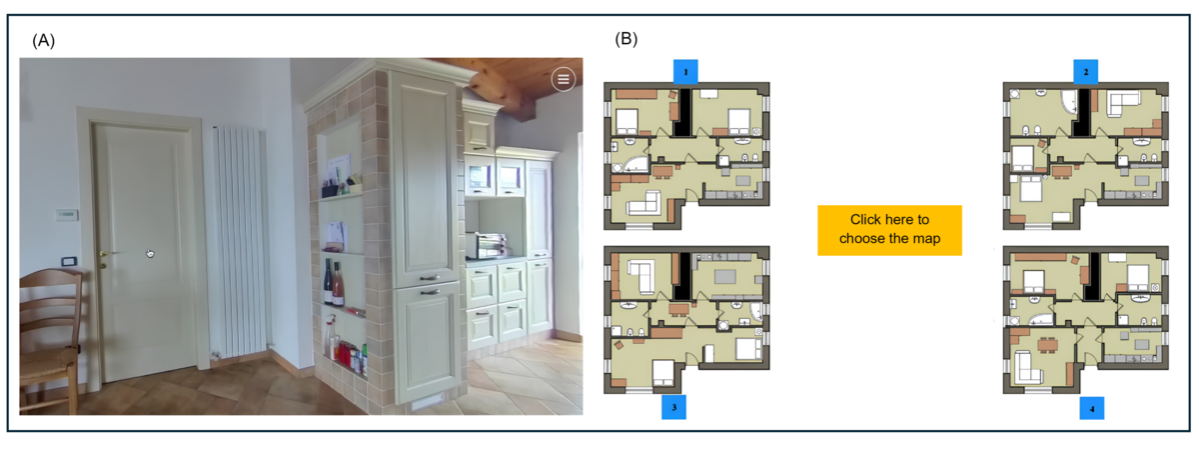
(A) The encoding phase: participants navigate in the 360° house by clicking on each door and (B) the retrieval phase: participants have to choose the right maps of the apartment.

This task takes place in a virtual home, in which users have to freely explore the diverse rooms and then identify the right map of the apartment (in an allocentric way). The residence encompasses a kitchen area that incorporates a centrally positioned table, along with intricate elements such as appliances, shelves, and furnishings. The spatial configuration of the apartment encompasses the living room with a primary ingress and a portal that grants access to a passageway. The corridor is bordered by 6 doors, each leading to separate rooms, including the living room, 2 bedrooms, and 2 bathrooms. Participants were presented with the instructions to enter the apartment and examine the various rooms while considering their spatial arrangement within the dwelling. The task starts from the dining room and kitchen area. The participant is afforded the freedom to engage in interactions by clicking on the different doors to transition between rooms. Once the participants believe they have completed their exploration of all the rooms, the clinician instructs them to return to the main room to continue with the testing process. Upon activating the main entrance, a set of instructions appears, presenting 4 distinct maps depicting the layout of the previously explored domicile. The request is to look through the available options and identify the appropriate one throughout the maps displayed on the screen in an allocentric perspective (Figure 1). The score is defined by the correct or incorrect response of the participant.

#### Episodic Memory Tasks

**Figure 2:**
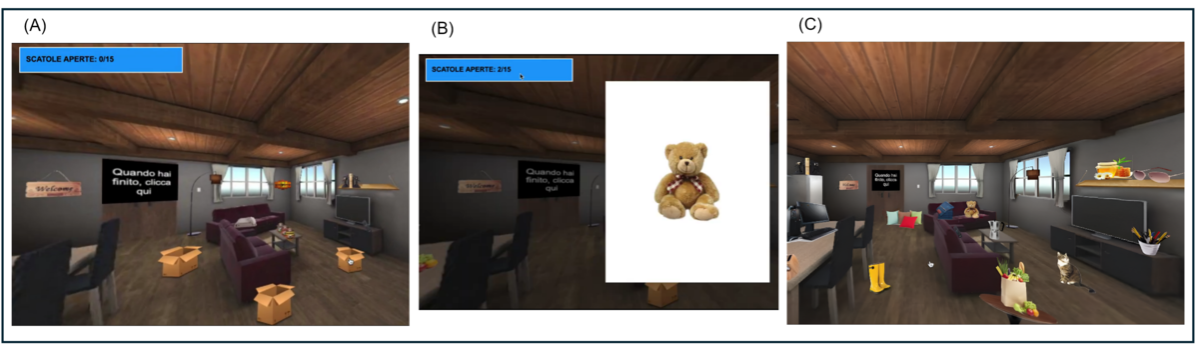
(A) The encoding phase: participants explore the 360° house and click on each box; (B) participants have to label the objects that appear; and (C) the retrieval phase: participants have to explore the previous environment and click only the objects they previously saw in the boxes.

This task is composed of a virtual house with several distinct areas. A total of 45 objects were generated to populate the virtual house and serve as either targets or distractors. Initially, 4 distinct semantic categories were identified: kitchen items, living room items, musical instruments, and home decor items. All the objects mentioned were tangible items that are typically encountered in residential dwellings. In the learning phase, the objective entailed the identification of 15 distinct entities enclosed within various boxes distributed in the house’s environments, with each object being easily recognizable. To carry out the task, participants were provided with instructions to open each of the boxes and verbally identify the objects contained within. Specifically, they were instructed to imagine themselves entering Marco’s new residence to aid him in relocating his stuff; this aspect was introduced to improve the ecological aspect. Among the 15 target objects, 15 distractors, which shared a semantic category with the target items, were strategically positioned in comparable locations. The task concluded upon the completion of opening all the boxes. At this point, it is advised that participants proceed to select the primary entrance of the house by clicking on the designated area, and a neutral gray environment is presented, accompanied by the following prompt: “Reflect upon the contents of the boxes that were previously opened and record any items that you remember.” This phase is referred to as the free recall phase. Following a 10-minute interval, the recalling phase takes place and the user (revisiting Marco’s residence) has to navigate the apartment and select objects that have been previously extracted from the boxes by tapping on them. Within the confines of the dwelling, one can observe the presence of various objects that are dispersed throughout the space. These objects are comprised of the contents of the boxes, as well as an additional set of 15 distractors that were initially introduced during the encoding phase and an additional set of new 15 objects, belonging to the same semantic categories as the distractors. Upon selecting an object, a luminous outline emerges, serving as a form of feedback for the user. Users can select and deselect objects. The task concludes when the participant indicates that they have chosen all the objects, selecting the primary entrance of the house by clicking on the designated area (Figure 2).

The scoring considers the number of objects freely recalled and the number of objects recognized during the recall phase. These are divided into target objects, distractors that were displayed during the encoding phase, and the new set of distractors.

Before spatial and episodic tasks, participants underwent a familiarization phase in which they were introduced to the virtual devices within a simulated household setting. During this phase, participants were instructed to navigate through the virtual environment and adhere to the provided instructions. According to the instructions provided in the tablet version, users are instructed to swipe their fingers horizontally and vertically. This directive is used to establish the comprehensibility of the environment’s navigability in a complete circle of 360°, as well as to indicate that the objects within said environment possess the capability of being interacted with through clicking. Moreover, participants are requested to select a door to move to a different room. This instruction serves to convey the concept of interactivity within the environment, emphasizing that the act of clicking on the doors enables the transition between rooms. This functionality is integral to the execution of the spatial memory task.

Each scene provided in the app allows users to look around while performing the tasks. At any time, the interaction is provided either through buttons superimposed on the video or through a specific user interface. Instructions are given to the user in the form of written text before the learning and recalling phases. In particular, the virtual environment refers to a tangible living space that has been captured through the use of the Insta360 One X 360° camera configuration.

SemAPP was developed thanks to a new ad hoc platform incorporating preexisting software capable of playing 360° media, in addition to JavaScript and HTML extensions. A group of clinicians and experts in UX participated in the development of the app. Tasks were chosen based on the most common issues in the literature regarding older people at risk of dementia [18,25]. Furthermore, we draw inspiration from numerous well-used tests to assess cognitive deficits in ecological settings [18] and adapt them to 360° technology.

### UX Measures

Quantitative and qualitative measures were captured following and during the experience with the app. We use the System Usability Scale (SUS) [26,27], the Senior Technology Acceptance Model (STAM) [28], the Independent Television Commission Sense of Presence Inventory (ITC-SOPI) [29], and the Thinking Aloud Protocol (TAP) [30].

SUS is a self-report tool, introduced by Brooke [26], which provides a global measure of the usability of a system, based on the following criteria: effectiveness (the ability of users to complete tasks using the system, and the quality of the output of those tasks), efficiency (the level of resource consumed in performing tasks), and satisfaction (users’ subjective reactions to using the system). The SUS is composed of 10 items, where the participants could indicate their degree of agreement through a 5-point Likert scale (from 1=strongly disagree to 5=strongly agree). SUS scores range from 0 to 100; the higher the score, the more the system reflects the criteria of effectiveness, efficiency, and satisfaction.

The STAM is a questionnaire that explores older people’s acceptance of technology and age-related health characteristics. The questionnaire used in this study is a shorter 14-item version of the original one, which aimed to predict older people’s multidimensional acceptance of everyday technology. In particular, the factors explored are attitude through technologies, perception of control, anxiety related to technologies, and general health status. For each area, participants must define their degree of agreement with some statements using a 10-point Likert scale ranging from 1=strongly disagree to 10=strongly agree. For each subscale the score ranges from 1 to 10; the higher the score more the results are satisfactory in terms of attitude, control, less anxiety, and health status.

The ITC-SOPI is a self-report scale that explores the individual’s feelings during the experience. The questionnaire is structured in parts A and B, which respectively investigate the user’s experience after and during the mediated environment. Participants must indicate their degree of agreement through a 5-point Likert scale (from 1=strongly disagree to 5=strongly agree). The specific factors explored through the 42 items of the questionnaire are the sense of physical space, engagement, ecological validity, and negative effects. Each subscale received a maximum score of 5; the higher the score, the better the result.

We use the TAP to investigate a wide range of mental processes and to analyze the cognitive problems people have with learning and using technology. The TAP is a qualitative instrument consisting of detailed observation and documentation of the users’ interaction with the proposed system. During the interaction, participants were asked to comment out loud about their thoughts, doubts, and expectations related to the characteristics of the experience. The real-time evaluation is important because it avoids relevant information being lost if it is collected after the experiment [31,32].

### Procedures

Participants took part in 2 different sessions, roughly 1.5 hours. The first one consists of a preliminary discussion with the user about the aim of the study, an informative consensus sign, gathering participant personal data (age, sex, and education) and their confidence with technology and electronic devices, and assessing their general cognitive state with a neuropsychological battery. Based on the results of the assessment phase, we assigned participants in one of the 2 experimental conditions: healthy individuals and patients with MCI. In the second phase, the UX measures were implemented. Participants were asked to accomplish the 2 tasks of semAPP in a randomized order. The only information given to participants was to read and follow the instructions on the screen. Experimenters provided more information when participants did not understand the instructions or if they had issues in using the device (according to the TAP). During the experiment, each issue related to the usability (observed by the experimenter or declared by the participant) of the app was reported on the protocol. After completing the tasks, the participants had to fill out questionnaires. We ran the app on an 11.2-inch iPad.

### Statistical Analysis

The statistical analysis was conducted using jamovi (version 2.5; jamovi Project). A significance level of *P*<.05 was established for all tests. In the initial phase, we conducted descriptive analyses on demographic and usability data for both the entire sample and each subgroup, namely the healthy control (HC) and MCI groups. Subsequently, parametric analyses, specifically the independent 2-tailed *t* test (also known as the Student *t* test), were performed to compare groups. Subsequently, we performed correlation analyses using the parametric Pearson test to examine the relationship between the virtual task experience, as evaluated by the SUS, and the 4 scales of the ITC-SOPI questionnaire, as well as the measures of older individuals’ acceptance of technology known as the STAM. We carefully considered the appropriateness of both parametric and nonparametric methods. To determine the most suitable approach, we initially conducted a graphical analysis of the data. Through this preliminary graphical examination, we observed that the data distribution closely approximates a normal distribution. Moreover, we performed the Shapiro-Wilk test to verify if our samples were from a normal distribution. This visual inspection and analysis suggested that the assumptions underlying parametric tests might be reasonable. Given these observations, we decided to proceed with parametric methods for comparing groups and correlation analyses. Parametric methods can be robust even with smaller samples if the data are approximately normally distributed. This allows us to take advantage of the greater statistical power offered by parametric tests.

## Results

Starting with qualitative data, all participants had some difficulties in comprehending the instruction during the familiarization phase. Certain individuals encountered challenges when attempting to engage with the tablet, specifically regarding the execution of finger-dragging gestures on the screen. A substantial number of users encountered difficulties while attempting to execute the exercises due to the challenging nature of exploring the environment, necessitating the need for additional support. Tables 1 and 2 show detailed results of the TAP for the 2 groups.

**Figure 3:**
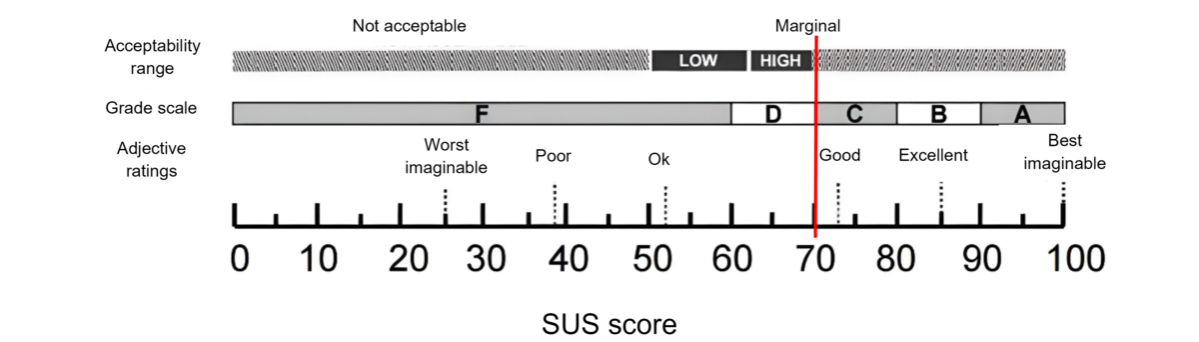
Graphical representation of the interpretation of SUS. The red vertical line indicates the mean score (70.22, SD 16.46), according to the rating comparison scale provided by Bangor et al. SUS: System Usability Scale.

Regarding the quantitative data, according to Bangor et al [27], the mean score of the SUS indicates that users perceived the app as having a good level of acceptability (mean 70.22, SD 16.46), as shown in Figure 3 [27]. There were no differences between groups.

The results of the STAM scale reveal that users have a positive attitudinal belief toward technology (mean 7.39, SD 2.16 out of 10), as well as a high level of control belief (mean 7.42, SD 2.06 out of 10). However, the MCI group presented a mean average score significantly higher than HC in both variables (*P*=.049 and *P*=.02, respectively). Users presented a medium level of anxiety related to technology (mean 5.81, SD 2.59 out of 10) and considered themselves in good health conditions (mean 7.78, SD 1.50 out of 10). As shown by the ITC-SOPI subscale investigating spatial presence, participants felt a generally low level of being there in the virtual environment (mean 2.82, SD 0.90 out of 5); they experienced a good level of engagement (mean 3.38, SD .64 out of 5) and naturalness of the environments (mean 3.58, SD 0.89 out of 5). Referring to the subscale of negative effects, all participants reported a low score of side effects (mean 1.57, SD 0.53 out of 5), indicating that the use of semAPP did not induce dizziness and cybersickness. Descriptive statistics for demographics and all UX questionnaires in the sample are shown in Table 3.

**Figure 4:**
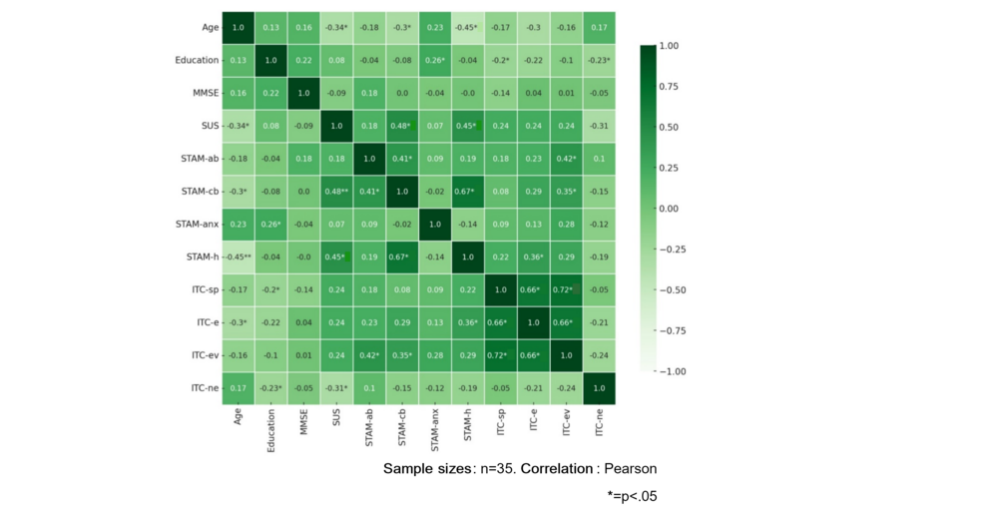
Graphical representation of the correlation matrix of the total sample. ITC-e: International Test Commission–Sense of Presence Inventory engagement subscale; ITC-ev: International Test Commission–Sense of Presence Inventory ecological validity subscale; ITC-ne: International Test Commission–Sense of Presence Inventory negative effects subscale; ITC-sp: International Test Commission–Sense of Presence Inventory spatial presence subscale; MMSE: Mini-Mental State Examination; STAM-ab: senior technology acceptance model attitude through technologies subscale; STAM-anx: senior technology acceptance model anxiety related to technologies subscale; STAM-cb: senior technology acceptance model perception of control subscale; STAM-h: senior technology acceptance model health conditions subscale; SUS: System Usability Scale.

**Figure 5:**
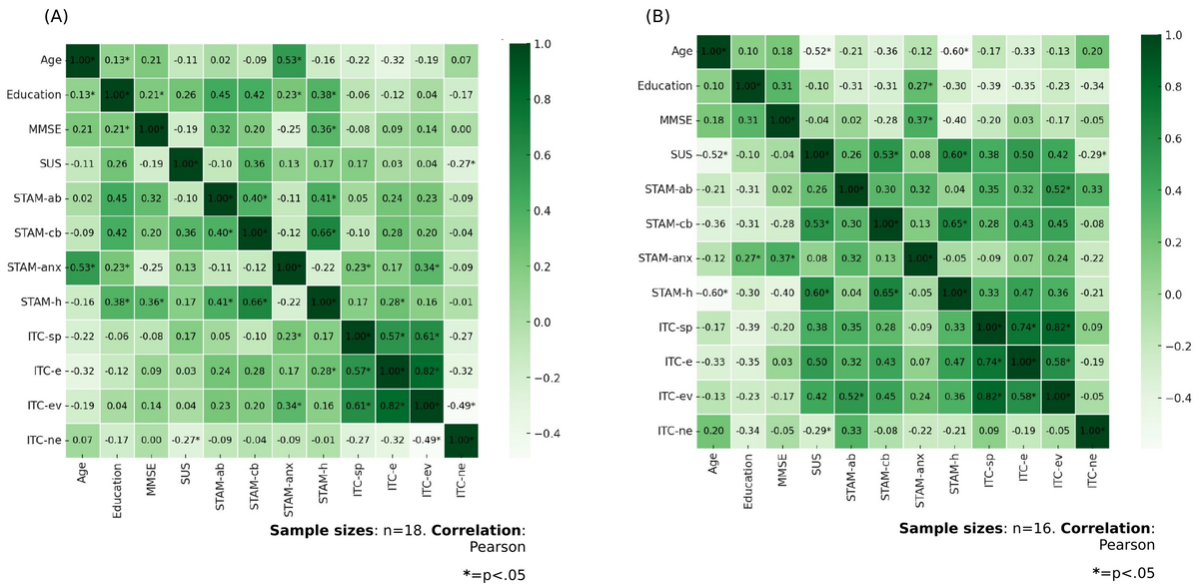
(A) The graphical representation of the correlation matrix of the HC group and (B) the graphical representation of the correlation matrix of the patients with MCI. HC: healthy control; ITC-e: International Test Commission–Sense of Presence Inventory engagement subscale; ITC-ev: International Test Commission–Sense of Presence Inventory ecological validity subscale; ITC-ne: International Test Commission–Sense of Presence Inventory negative effects subscale; ITC-sp: International Test Commission–Sense of Presence Inventory spatial presence subscale; MCI: mild cognitive impairment; MMSE: Mini-Mental State Examination; STAM-ab: senior technology acceptance model attitude through technologies subscale; STAM-anx: senior technology acceptance model anxiety related to technologies subscale; STAM-cb: senior technology acceptance model perception of control subscale; STAM-h: senior technology acceptance model health conditions subscale; SUS: System Usability Scale.

Moreover, we analyze the correlations between older adults’ experience with the virtual assessment task, as measured by the SUS and the ITC-SOPI, and their attitudes and willingness to use the technology, as assessed by the STAM. The objective is to determine whether older adults who exhibit more favorable attitudes toward technology use also perceive the virtual assessment task as more usable and user-friendly (as indicated by SUS scores) and experience a greater sense of presence and immersion during the task (as indicated by ITC-SOPI scores). Considering the total sample, SUS positively correlated with the STAM perception of control (*r*=0.48; *P*=.004) and STAM health conditions (*r*=0.45; *P*=.008) scales: those who perceive the virtual assessment task to be more usable and user-friendly might also more likely to feel a sense of control over the technology they are utilizing and a more positive perception of their health. When we consider separately the 2 groups, the correlation between the SUS and STAM remains in the MCI group, but not in the HC group. Furthermore, there was a positive correlation observed between the ecological validity scale of the ITC-SOPI and the attitudinal beliefs (*r*=0.35; *P*=.04) and control beliefs (*r*=0.42; *P*=.02) subscales of the STAM . Additionally, a positive correlation was found between the STAM health conditions subscale and the engagement subscale of the ITC-SOPI (*r*=0.36; *P*=.04). The observed correlation suggests that individuals who obtained higher scores on the STAM health conditions subscale, which reflects their perception of their health and well-being, demonstrated a greater likelihood of experiencing heightened levels of engagement within the virtual environment. In other words, participants who possessed the belief that the utilization of technology had positive impacts on their well-being exhibited higher levels of immersion and engagement in the virtual experience. This is true when we consider the MCI group alone, not for the HC group. Correlations for the entire sample and each group are shown in Figures 4 and 5.

**Table 1:** Qualitative usability results of the Thinking Aloud Protocol in the HC^a^ group (n=16).

Task		Problem	Solution	Participants, n (%)
**Familiarization**				
	**Reading the instructions**	—^b^	—	—
	**Comprehension of the instructions**	Difficulties in understanding the instructions concerning the direction of exploration	Clarify the instructions	15 (94)
	**Device interaction**			
		Instructions do not appear in a functional position to read on the screen	Insert the instructions in the middle of the screen	2 (13)
		Difficulties with dragging the finger on the screen	Provide a previous tutorial on using the touchscreen	2 (13)
	**Execution**	—	—	—
**Task 1: figure recognition**				
	**Reading the instructions**	Unread instructions	Insert auditory instructions; provide a button to begin the exercise that appears after a few seconds	1 (6)
	**Comprehension of the instructions**	Difficulties in understanding the instructions for selecting the items	Improve the quality of the instructions	1 (6)
	**Device interaction**	—	—	—
	**Execution**			
		Difficulties in exploring the environment	Insert a more functional hotspot; provide more precise instructions to complete the task	10 (63)
		Item selected after operator suggestion	Insert a more functional hotspot; provide more precise instructions to complete the task	2 (13)
		Difficulties in understanding how to finish the exercise during the Encoding phase	Insert a more functional hotspot; provide more precise instructions to complete the task	3 (19)
		Difficulties in selecting desired items during the Recognition phase	Improve the target’s dimension; spacing the targets further apart; implement the quality of the video	1 (6)
**Task 2: spatial memory task**				
	**Reading the instructions**	—	—	—
	**Comprehension of the instructions**	Difficulties in understanding the instructions	Clarify the instructions	1 (6)
	**Device interaction**	—	—	—
	**Execution**			
		Difficulties in understanding how to finish the exercise	Insert more specific instructions; identify an ad hoc hotspot that indicates in a functional way how to exit	2 (13)
		Difficulties in selecting the hotspot to finish the exercise	Extend hotspots throughout the target	1 (6)
		Too many steps in the map selection process	Provide a lean selection process; delete unnecessary steps	5 (31)
		Difficulty in exploring the environment without operator support	Insert a more functional hotspot	2 (13)

^a^HC: healthy control.

^b^Not available.

**Table 2:** Qualitative usability results of the Thinking Aloud Protocol in the MCI^a^ group (n=18).

Task		Problem	Solution	Participants, n (%)
**Familiarization**				
	**Reading the instructions**	—^b^	—	—
	**Comprehension of the instructions**	Difficulties in understanding the instruction concerning the direction of exploration	Clarify the instructions	12 (67)
	**Device interaction**			
		Instructions do not appear in a functional position to read on the screen	Insert the instructions in the middle of the screen	1 (6)
		Difficulties with dragging the finger on the screen	Provide a previous tutorial on using the touchscreen	1 (6)
		Difficulties in clicking on the screen	Provide a previous tutorial on using the touchscreen	1 (6)
	**Execution**			
		Difficulties in exploring the environment	Provide more specific or intuitive instructions to explore; provide a more functional hotspot	5 (28)
		Difficulties in finding the items	Provide more specific or intuitive instructions to explore; provide a more functional hotspot	2 (11)
		Difficulties in finding the hotspot to move forward to the next environment	Provide more specific or intuitive instructions to explore; provide a more functional hotspot	3 (17)
**Task 1: figure recognition**				
	**Reading the instructions**	Unread instructions	Insert auditory instructions; provide a button to begin the exercise that appears after a few seconds	1 (6)
	**Comprehension of the instructions**	Difficulties in understanding the instructions	Insert auditory instructions; provide a button to begin the exercise that appears after a few seconds	1 (6)
	**Device interaction**	—	—	—
	**Execution**			
		Difficulties in exploring the environment	Provide more specific or intuitive instructions to explore; provide a more functional hotspot	10 (56)
		Difficulties in selecting desired items during the recognition phase	Improve the target’s dimension; spacing the targets further apart; implement the quality of the video	1 (6)
**Task 2: spatial memory task**				
	**Reading the instructions**	—	—	—
	**Comprehension of the instructions**	Difficulties in understanding the instructions	Insert auditory instructions; clarify the instructions	1 (6)
	**Device interaction**	Difficulties in selecting the items to provide the answer	Insert a tutorial; improve the dimensions of the buttons	1 (6)
	**Execution**			
		Difficulties to explore the environment without operator support	Implement the house exploration in a tutorial	6 (33)
		Too many steps in the map selection process	Provide a lean selection process; delete unnecessary steps	3 (17)

^a^MCI: mild cognitive impairment.

^b^Not available.

**Table 3:** Descriptive statistics for demographics and UX^a^ questionnaires.

	Total sample (N=34), mean (SD)	MCI^b^ (n=18), mean (SD)	HC^c^ (n=16), mean (SD)	Student *t* test (*df*=32)	*P* value
Age (years)	74.65 (7.64)	73.44 (7.32)	76.00 (8.01)	0.972	.34
Education	13.62 (4.24)	13.33 (4.43)	13.94 (4.14)	0.409	.68
ADL^d^	5.82 (0.58)	5.94 (0.24)	5.69 (0.79)	–1.313	.20
MMSE^e^	27.07 (1.69)	27.31 (1.97)	26.80 (1.31)	–0.871	.39
SUS^f^	70.22 (16.46)	73.61 (16.32)	66.41 (16.29)	–1.286	.21
STAM-ab^g^	7.39 (2.16)	8.07 (1.36)	6.62 (2.65)	–2.051	.049^h^
STAM-cb^i^	7.42 (2.06)	8.18 (1.11)	6.57 (2.54)	–2.452	.02^h^
STAM-anx^j^	5.81 (2.59)	5.44 (2.58)	6.22 (2.61)	0.868	.39
STAM-h^k^	7.58 (1.50)	7.90 (0.99)	7.22 (1.88)	–1.328	.19
ITC-sp^l^	2.82 (0.90)	2.71 (0.86)	2.94 (0.96)	0.752	.46
ITC-e^m^	3.38 (0.64)	3.31 (0.58)	3.46 (0.71)	0.682	.50
ITC-ev^n^	3.58 (0.89)	3.61 (0.79)	3.54 (1.02)	–0.243	.81
ITC-ne^o^	1.57 (0.53)	1.46 (0.481)	1.69 (0.58)	1.268	.21

^a^UX: user experience.

^b^MCI: mild cognitive impairment.

^c^HC: healthy control.

^d^ADL: activity of daily life.

^e^MMSE: Mini-Mental State Examination.

^f^SUS: System Usability Scale.

^g^STAM-ab: Senior Technology Acceptance Model attitude through technologies subscale.

^h^Significant group difference between HC and MCI.

^i^STAM-cb: Senior Technology Acceptance Model perception of control subscale.

^j^STAM-anx: Senior Technology Acceptance Model anxiety related to technologies subscale.

^k^STAM-h: Senior Technology Acceptance Model health conditions subscale.

^l^ITC-sp: International Test Commission–Sense of Presence Inventory spatial presence subscale.

^m^ITC-e: International Test Commission–Sense of Presence Inventory engagement subscale.

^n^ITC-ev: International Test Commission–Sense of Presence Inventory ecological validity subscale.

^o^ITC-ne: International Test Commission–Sense of Presence Inventory negative effects subscale.

## Discussion

### Principal Findings

It is necessary to measure cognitive processes in a way that is similar to what happens in real life to provide functional feedback that accurately reflects the patient’s capacity to respond to daily problems. In this study, a novel tablet-based app with 360° media has been developed to solve the issue of the ecological validity of the currently available test, which used abstract settings. With the increasing scientific discussions supporting the use of new technologies in neuropsychological assessment [33], semAPP was developed using 360° content to simulate real-world circumstances.

The idea behind this app was inspired by the evidence that virtual environments, recreating real situations, might enhance the engagement and pleasure of users, as well as measuring ability that reflects what happens in daily life. As cognitive evaluation increasingly relies on immersive technology, it is crucial to develop user-friendly programs that enable academics, physicians, and patients without engineering skills to access cognitive activities. Attention in their design and execution is a critical issue if one wishes to ensure task reliability and validity. Thus, the final users’ experience is an essential element in designing instruments that reflect population needs and expectations. On these bases, a team of experts took part in the design of the app, and the prototype was tested on a group of final users. Our data promise to provide an app usable and accepted by users. SemAPP was judged as an easy-to-use tool for all participants who could interact with it independently under the supervision of clinicians; users evaluated semAPP to be usable and they were willing to use it. They were satisfied and expressed interest in using it. The users were fascinated by using the tablet to explore the virtual environment and enjoyed performing exercises in this innovative way. They were also able to accomplish all expected goals without learning a lot of processes or complex actions; they were at ease using the device because they were familiar with it, and many of them claimed to do so frequently. This is in line with the core features of satisfaction, effectiveness, and efficiency used to define usability [26]. Moreover, users who perceived the virtual assessment task to be more usable and user-friendly have also a positive perception of health and a sense of being able to use apps successfully and effortlessly. Our results are independent of groups; this is promising for the future use of semAPP, which is in line with the needs and expectations of a healthy and aging population with MCI. Thus, it could be used effectively and efficiently by different pools of people.

The results derived from the users’ ideas about technology show that all users have a positive appraisal of using technology and they believe that using it would be beneficial for their quality of life. They also believe that using the proposed technology might be free of effort and feel a sense of being able to use it successfully. Contrary to our expectations, these features are significantly higher in the MCI group. The results could be explained by the clinical condition. Patients with MCI are conscious of their deficits, and they could be more engaged in having positive feelings with the app to improve their abilities [34]. All users experience a sense of apprehension when they are faced with the possibility of using technology, for example, due to the fear of making some mistake; this is common in aging [35]. Additionally, older people feel themselves in a good health condition related to biological, psychological, and social capabilities, which decrease with aging (ie, as age increases, there is a decrease in the perception of good health) [35].

Investigating the experience with the virtual scenes, participants reported that the app offered them a sense of engagement and the natural environment, as shown by the ITC-SOPI. However, they detected a generally low level of being in the virtual environment, which may be attributed to the low degree of immersion offered by the tablet. We chose the tablet because it can be used more quickly and easily than other tools for reproducing 360° media, even without the guidance of a therapist or caregiver. However, the media were deployed on a flat screen in a less ecological situation than a head-mounted display, for example. This feature may influence the feeling of immersion [14]. On the other hand, side effects and cybersickness were minimal.

Our findings also support literature that suggests that engagement and a sense of ecological validity improve when users have expectations of positive feelings in using technology, potentially beneficial effects for quality of life, no effortlessness, and a sense of being able to use technology successfully [36,37]. In other words, users’ ideas are closely related to how they encounter and evaluate experiences, reflecting anticipated behavior, direct attention, and interpretation, influencing the perceptions of the product [38]. Likewise, older adults who believed that the use of technology had positive impacts on their well-being exhibited higher levels of immersion and engagement in the virtual experience. These results reinforce how a greater likelihood of experiencing heightened levels of engagement within the virtual environment is linked to personal expectations.

Some technical and interface problems were brought up by the TAP during the study, and it is possible that these issues could be resolved by making the instructions clearer and adding the possibility of hearing them. To solve the issues related to interacting with the tablet, we could add a specific training phase focused on the technical use of the device. Thanks to these upgrades, the app should be easier to use.

Nevertheless, while several virtual reality instruments exist to assess memory, only a limited number leverage the unique advantages of 360° media, using computer-generated scenarios [39]. Additionally, some of these tools are delivered using sophisticated solutions, such as head-mounted displays, which can present significant barriers to adoption among elderly individuals [11,40]. Older adults often hesitate to use such advanced technology due to various perceived obstacles [2]. In contrast, semAPP is a highly customizable app designed to be delivered through a range of devices, from smartphones to head-mounted displays. This flexibility offers a more accessible and user-friendly solution that accommodates the varying technological comfort levels of older adults, striking a balance between environmental control and realism.

Given the promising data and the potential of semAPP, further research is essential to fully integrate the app into existing health care frameworks. Integrating our app into these systems would streamline the collection and accessibility of data, enhancing the overall efficiency of cognitive assessments. Additionally, by facilitating remote evaluations through telemedicine, semAPP can become a usable and valuable tool for managing and monitoring cognitive health in aging populations.

### Limitations

This work is not exempt from limitations. The market for 360° devices currently offers a few benefits that might give the measurements obtained a higher ecological worth. However, because of the lack of active navigation and the restricted opportunity for engagement within the surroundings, the low degree of immersion that 360° media distributed with tablet exhibits represents one of their most significant drawbacks. In the given context, this can be detrimental in terms of involvement and emotions of naturalness. However, for older people who lacked the necessary abilities to work with more sophisticated technologies, the absence of these characteristics might have been seen as a benefit rather than a limitation.

Despite the widespread adoption and user-friendly nature of tablets as a technological solution, potential biases and variances may arise due to differing levels of familiarity and comfort with technology among participants. While many participants reported frequent tablet use and demonstrated familiarity with the device, they might initially encounter challenges with the 360° media, which possesses distinct characteristics compared to traditional media. These challenges could indicate that differences in technological familiarity can introduce biases and variances in the data, potentially affecting the validity of the findings. Although the participants were able to accomplish all expected goals, we will incorporate a familiarization phase before the test to mitigate these limitations. This phase is designed to acclimate participants to the specific media, ensuring they have sufficient exposure and comfort with the new media format, thereby standardizing their level of comfort and familiarity.

Taking our sample size into account is essential. Based on prior research that used a small number of participants to measure usability [41], as also highlighted by Virzi [42], we chose to use a small number of users. Most usability issues, according to the researchers, are discovered in 4 to 5 individuals, who are progressively less likely to divulge fresh information. We chose 34 users to represent a range of demographics, technical proficiency, and technology knowledge; yet many of them noted the same usability issue at the TAP.

Finally, the heavy reliance on self-reported data may introduce subjective biases. Implementing additional objective measures, while beneficial, is challenging with an older adult population and limits capturing UX in a manner that is both simple and well-accepted. Nonetheless, future research should consider integrating objective measures to complement self-reported data providing a more comprehensive assessment.

Even with its limitations, these findings support the usability of 360° assessment, implementing the objective evaluation of ecological situations. However, additional work will be implemented to improve the problems revealed by the TAP and to explore the validity of the instrument.

### Conclusions

We developed a new 360° tool to assess memory in aging, and we explored its usability and the correlated experience in using it. We focused on 2 different populations: healthy older adults and patients with MCI, to examine possible differences in terms of usability and, thus, to verify if our app was designed to be used by both target user groups excellently. Our results were satisfactory, showing the achievement of goals relating to the possibility of using the app by the target sample, with a positive experience.

Our research also supports the importance of a user-centered approach that adapts the app to the target population, analyzing the needs and clinical conditions of older people. To achieve this goal, we require a team of clinicians who have clinical competencies and UX experts. They designed an app focused on the clinical features of patients, in line with the major requirement to create the best possible experience in terms of usability and accessibility of the app. Our findings support the idea that users’ thoughts about technology might influence how the product is seen, as well as the experience in the virtual environments (ie, the engagement and sense of being there). Given the complexity of human experiences, the usability and effective use of an instrument potentially depends on a great variety of parameters. The experience presupposes that all personal and technological factors are connected, interact, and change one another and the experience is what comes out of this process [36]. In this panorama, evaluating the product while considering end users could be crucial. Researchers must be aware that, depending on the personal experience and clinical condition at hand, users can have different experiences with the same product [43]. Indeed, if we do not consider the end user’s experience, there may be a potential mismatch between the designers’ intentions and users’ actual anticipation.

## Abbreviations

HC: healthy control
IRCCS: Istituto di Ricovero e Cura a Carattere Scientifico
ITC-SOPI: Independent Television Commission Sense of Presence Inventory
MCI: mild cognitive impairment
semAPP: spatial and episodic memory assessment application
STAM: Senior Technology Acceptance Model
SUS: System Usability Scale
TAP: Thinking Aloud Protocol
UX: user experience
